# The effect of life skills training on reducing domestic violence and improving treatment adherence in women with diabetes experiencing intimate partner violence: a randomized clinical trial based on the theory of self-efficacy

**DOI:** 10.1186/s12889-024-20913-y

**Published:** 2024-12-05

**Authors:** Shahrbanoo Rezaee, Razieh Bagherzadeh, Mahnoush Reisi, Leila Fotovat, Hakimeh Vahedparast

**Affiliations:** 1 Student Research Committee, Faculty of Nursing and Midwifery, Bushehr University of Medical Sciences, Bushehr, Iran; 2 Department of Midwifery, Nursing and Midwifery Faculty, Bushehr University of Medical Sciences, Bushehr, Iran; 3 Department of Health Education and Health Promotion, Faculty of Health, Bushehr University of Medical Sciences, Bushehr, Iran; 4 Student Mental Health Counseling Office, Student Cultural Vice-Chancellor, Bushehr University of Medical Sciences, Bushehr, Iran; 5 Department of Medical-Surgical Nursing, Nursing and Midwifery Faculty, Bushehr University of Medical Sciences, P.O.Box. 7518759577, Salmane-farsi Blvd, Bushehr, Iran

**Keywords:** Intimate partner violence, Type 2 diabetes mellitus, Life skills training, Self-efficacy, Women

## Abstract

**Background:**

Intimate partner violence (IPV) is a global health problem and the cause of chronic diseases, such as diabetes. It has a negative effect on adherence to treatment, decreases self-efficacy beliefs, and intensifies stress in women. Therefore, this study aimed to investigate the effect of life skills training based on the self-efficacy theory on IPV and adherence to treatment in women with type 2 diabetes.

**Methods:**

This trial was conducted using a pretest-posttest design and follow-up after one month. The samples included 100 women selected by convenience sampling with random block allocation with type 2 diabetes and IPV. The intervention consisted of 8 sessions over one month of life skills training based on self-efficacy theory. Participants completed questionnaires at pre-test, post-test and follow-up, including a demographic information form and questionnaires on IPV and treatment adherence. Considered statistically significant at *P* < 0. 05.

**Results:**

The mean changes in IPV scores from the pre-test to the post-test were − 8.38 ± 4.06 and − 0.06 ± 3.09 in the intervention and control groups, respectively. Also, the reduction in the intervention group was significantly more than in the control group (*P* < 0.001; 95%CI=-9.75; -6.89). The mean changes in IPV scores from post-test to follow-up were − 1.36 ± 3.47 and 1.50 ± 4.14 in intervention and control groups, respectively, indicating a statistically significant difference between the two groups (*P* < 0.001; 95%CI=-4.38; -1.34). The mean changes in adherence scores from the pre-test to the post-test were 11.40 ± 4.23 and 0.68 ± 3.49 in the intervention and control groups, respectively. The increase was significantly higher in the intervention group than in the control group (*P* < 0.001; 95%CI = 9.18; 12.26). The mean changes in adherence scores from post-test to follow-up were 2.68 ± 5.06 and − 0.86 ± 2.43 in the intervention and control groups, respectively. The difference between the two groups was statistically significant (*P* < 0.001; 95%CI = 1.95; 5.12).

**Conclusion:**

Life skills training based on self-efficacy theory reduced IPV and improved treatment compliance in women with diabetes under IPV. It is recommended that this training be taught to other patients with chronic conditions as a means of violence prevention and treatment adherence.

**Trial registration:**

The trial was registered with the Iranian Registry of Clinical Trials (IRCT) on 13 October 2022 and can be found on the Iranian Registry of Clinical Trials platform. IRCT registration number: IRCT20090522001930N6.

## Background

Intimate partner violence (IPV) represents the most prevalent and pervasive form of violence against women globally [1]. The term IPV is defined as any act perpetrated by a current or former male intimate partner in the context of marriage, cohabitation, or any other formal or informal union that results in physical, sexual, or psychological harm. It is the most prevalent and pervasive form of violence against women on a global scale [2, 3]. The World Health Organization (WHO) estimates that over a third of all women worldwide are affected by IPV, which requires an urgent and effective response [2]. The findings of a study conducted in Iran indicated that 98.8% of women had experienced at least one form of IPV [3]. Lye et al. reported that a mere 0.8% of Iranian women surveyed had disclosed instances of violence perpetrated against them by family members before their marriage. In contrast, 32.1% of the participants reported experiencing IPV after marriage [1].

IPV is a public health problem that has short- and long-term consequences on women’s physical and mental health [4]. IPV can lead to mental disorders, such as anxiety, depression, and fear or physical disorders, such as chronic pain, digestive disorders, migraine headaches, and pelvic pain [5], and exacerbate underlying chronic diseases, such as diabetes [4–6]. Physiological studies have confirmed that IPV causes diabetes by exacerbating stress and depression; the stress caused by IPV in women affects the hypothalamus-pituitary-adrenal axis, thereby increasing cortisol secretion and resistance of cells to insulin and causing diabetes [7]. Weitzman and Goosbi (2021) demonstrated that women who had experienced severe IPV during their lifetime exhibited a 33–200% increased likelihood of developing diabetes, cardiovascular disease, thyroid disorders, and cancer compared to women who had not experienced violence [6]. Other studies also show that anxiety and stress caused by the violence of the spouse can affect the exacerbation of type 2 diabetes mellitus (T2DM) [5–8].

Diabetes is a chronic disease whose prevalence is increasing globally; By 2050, the prevalence of diabetes is projected to reach 1.31 billion (1.22–1.39) cases globally [9]. This disease, which has physical, economic, and social complications, is incurable, but self-care and adherence to treatment contribute to its control. More than 95% of treatment for T2DM is done by the patient and by adhering to the treatment [10, 11].

Adherence can be defined as the level of concordance between an individual’s actions and the recommendations provided by a healthcare provider, particularly in relation to medication, dietary regimens, and lifestyle modifications [12]. It is a factor affecting the survival of patients with diabetes and their quality of life. However, studies show that adherence to treatment is not proper in patients with diabetes. A study by Orai et al. (2022) showed that more than two-thirds of patients with diabetes have an uncontrolled disease [13]. A set of factors, and not a single factor, determines the ability of patients to correctly follow treatment recommendations. Interpersonal interactions and the amount and type of support received are among the factors influencing treatment adherence [14]. The support of spouses can play a significant role in adherence to the recommended treatment regimens [15]. Positive spousal support is of great importance in maintaining treatment adherence behaviors and effective glycemic control in patients with diabetes [14, 15]. Haines et al. (2018) showed that treatment adherence among individuals with T2DM who received spousal support was significantly higher than that of those who did not [15]. In their review, Pratiwi et al. (2024) asserted that spouses play a pivotal role in improving the condition of patients with diabetes. Therefore, the marital relationship should be considered a significant factor in the couple’s health and disease management. If chronic disease is considered a shared problem of couples, the coping mechanisms exhibited by the spouse can prove an effective means of alleviating the distress experienced by the patient. Implementing positive coping strategies by the spouse can assist in reducing the patient’s distress, and joint efforts can facilitate disease management and enhance the patient’s self-efficacy [14]. Therefore, it can be posited that the behavior of the spouse of a woman with diabetes who is violent in nature will raise the stress levels of the patient. Consequently, it will affect the severity of T2DM and the level of adherence to the prescribed treatment.

The effects and consequences of each stressful event are determined based on how people deal with that event [16]; thus, appropriate coping skills are necessary to deal with a stressful event such as IPV and to adapt to different life situations. Behavioral science experts believe that one of the most effective programs to help people live a better and healthier life is the life skills training program [17].

Life skills are a set of abilities, knowledge, and behaviors that help people adapt to different life situations, face different cultures and environments, and contribute to mental health [17, 18]. Life skills training is a behavioral approach that seems to be suitable for all people who have problems expressing themselves in certain situations [17]. The lack of awareness about proper communication and social skills in women, who are the main pillars of the family, leads to low self-esteem and self-confidence, anxiety, and negative behaviors at home, which cause violent behavior in their spouses [17]. Sohrabzadeh et al. (2020) also showed that women’s lack of awareness about life skills is one of the factors affecting violence against them [19]. Life skills give people a sense of self-efficacy and control from within [17].

The theory of self-efficacy can be used to teach life skills effectively. According to Albert Bandura, who introduced self-efficacy theory in 1997 [20], people with strong beliefs about their abilities, compared to people who doubt their abilities, are more diligent and persistent in doing homework. As a result, their performance in doing homework is better. A high level of general self-efficacy can increase a person’s resilience against failures and disappointments [21]. This theory has received considerable attention as a predictor of behavioral changes and self-care management in educational and health research [22]. Research has shown that self-efficacy is related to IPV such that the lower the self-efficacy, the higher the perceived IPV [23]. As previously stated, low self-efficacy is associated with a greater perceived incidence of IPV, which may impede a patient’s capacity to adhere to their diabetes treatment adheres to the prescribed treatment plan. Furthermore, evidence suggests that life skills training is an efficacious method for facilitating adaptation and reducing violence against healthy women subjected to IPV [17, 18]. In light of the aforementioned evidence, the present study was designed to investigate the impact of life skills training based on self-efficacy theory on the IPV and the enhancement of treatment compliance in women with T2DM.

## Methods

### Design

The current study was a randomized controlled clinical trial with a pre-test, post-test, and follow-up design conducted in 2022–2023. All steps of the study have been done according to Declaration of Helsinki and the instructions of the Ethics Committee of Bushehr University of Medical Sciences (IR.BPUMS.REC. 1401.063) and Iranian Registry of Clinical Trials (IRCT20090522001930N6) in a single-blind manner. In this Study, informed consent to participate obtained from participants.

### Patient population and sampling

The study population included all women with diabetes experiencing IPV who visited hospitals, cardiologists, endocrinologists, internal medicine doctors’ offices, diabetes clinics, and comprehensive health service centers in Bushehr, Iran. Samples that met the inclusion criteria were invited to participate in the study by telephone.

According to the study by Mohammad-Beigi et al. [24] and taking into account the type I error, i.e., alpha of 0.05 and a power of 80%, the sample size was determined as 45 people in each group. Taking into account a 10% attrition rate, a sample of 50 people in each group was determined. The participants were randomly divided into the intervention and control groups. The recruitment phase began on 16 October 2022 and ended on 25 November 2022. Data collection was completed on 5 February 2023.

A total of 300 patients were screened, with 158 meeting the inclusion criteria and thus being eligible for further participation in the study. Of these people, 42 declined to participate in the study. Therefore, 100 people consented to participate and were randomly assigned to one of the two study groups (each comprising 50 participants) through block randomization. This randomization involved using 12 blocks of 8 and 1 block of 4 to classify the samples. In the initial phase, eight-block samples were created using two distinct groups. The study employed two distinct groups: Group A (the intervention group) and Group B (the control group). Each group comprised 70 unique modes. A total of 12 blocks were randomly selected from the entire set. The eight blocks were selected according to the order of letters, thus forming two groups, with four chances in each. Furthermore, four modes were prepared, and one block was randomly selected from among them. Subsequently, this block was distributed between the two groups, with each group afforded two chances.

### Inclusion and exclusion criteria

The inclusion criteria were as follows: women aged between 30 and 70 years, diagnosed with T2DM, willing to participate in the study, married and living with their spouse, and experiencing IPV, with at least six months passed since contracting the disease, with full hearing and speaking abilities, and with a minimum level of literacy. In this respect, experiencing IPV is defined as having at least one item from the IPV questionnaire, with a frequency of occurrence ranging from “often” to “always”.

The exclusion criteria were participation in life skills training courses, education in psychology or counseling, suffering from neurological disorders, psychiatric disorders (according to the self-report), a chronic disease that required special care (hemodialysis, heart failure, blood disorders, etc.), or withdrawing from the study.

### Intervention

After obtaining the code of ethics from Bushehr University of Medical Sciences, registering the trial, and receiving a clinical trial code (IRCT), coordination was made with the Vice-President of Treatment and the Vice-President of Health. The study’s objectives and methodology were initially delineated for the women who met the inclusion criteria, after which they provided consent to participate. The participants were informed that they could withdraw from the study at any phase of the research process. Furthermore, when obtaining informed consent, the participants were informed that the training would be conducted in a group setting. Should they feel uncomfortable in the presence of a familiar individual, they were granted the option to withdraw from the training group.

After completing the written informed consent, the demographic information form, and the IPV questionnaire, the procedure was followed in compliance with the ethical and safety recommendations of the WHO for research on IPV. Only those samples that had selected at least one item from the IPV questionnaire, with a frequency of occurrence ranging from “often” to “always”, were included in the study. The data was collected by the first author. They were then randomly assigned to control and intervention groups, with the educational intervention delivered face-to-face and as a group to the intervention group. The intervention group was divided into two groups of 25 individuals to observe the maximum social distance required to prevent the transmission of common respiratory diseases in the fall (e.g., colds and influenza) and to create an opportunity for greater interaction and discussion among participants. The location deemed most suitable for training the samples was selected based on its accessibility to facilitate the participation of the relevant samples. The training session was conducted in the conference room of the Bushehr Diabetes Clinic, which was equipped with a whiteboard, a video projector, and a computer projector. The seating was arranged in a U-shaped configuration, facilitating interaction between participants and the instructor. A telephone call was made to invite the participants to participate in the life skills training program. The intervention was administered in the form of eight two-hour life skills training sessions based on self-efficacy theory principles (Table 1). The mentioned intervention was conducted on a biweekly basis over four weeks. The intervention was delivered by a senior clinical psychologist experienced in teaching life skills under the supervision of the corresponding author to ensure that the intervention was delivered according to protocol. A list was prepared for each day, and all participants were recorded in all sessions. To maintain confidentiality, the following was explained to the samples: The samples were emphasized not to talk about the problems expressed outside the group. Each participant was given a pseudonym to avoid identification. It was also emphasized that samples could withdraw from the study at any time. The content of the life skills training programme was based on the guidelines recommended by the Iranian Ministry of Health and Medical Education in the book “Life Skills Training” authored by Fatta et al. (2015). The content was approved by relevant professors (two psychologists skilled in life skills training).

**Table 1:** Life skills training sessions

Session	Topics	Assignments
First session	Introducing, motivating, reviewing meeting structure and key rules, setting objectives and planning training	Writing feelings
Second session	Effective communication skills - effective interpersonal relationships	Determining the steps to achieve trust
Third session	Dominant experience strategy	Writing down your physical experiences when angry
Fourth session	The skill of bold behavior	Writing down your experiences while being exposed to aggressive behaviors
Fifth session	The skill of dealing with negative emotions	Identify negative thoughts
Sixth session	Stress management skills	Writing down times of stress
Seventh session	Time management skills	Writing down how you spend your time
Eighth session	Problem-solving skills	Writing down one of the recent problems in detail

The life skills program was based on Bandura’s proposed strategies for improving self-efficacy. These strategies included activities related to performance accomplishment, vicarious experience, verbal persuasion, and physiological states [25].

#### Performance accomplishment

The first self-efficacy strategy, that of dominant experiences, posits that self-efficacy is enhanced by successful experiences and diminished by unsuccessful ones. The training sessions emphasized the importance of following a step-by-step approach. The preparation for smaller and more attainable goals was conducted by involving samples to facilitate achieving larger goals. An action plan was devised to facilitate the desired behavior with the aid of achievable goals. They were requested to communicate these achievements, even if they are relatively minimal or inconsequential.

#### Vicarious experience

Witnessing the achievements of others, particularly individuals who share similar conditions and characteristics, has been demonstrated to foster a sense of self-efficacy. In this study, participants who had successfully implemented the recommended strategies were invited to share their experiences with others to provide a basis for further learning. This issue reinforces the belief in others that if others have been able to perform the desired behavior, they are also capable of doing so.

#### Verbal persuasion

Another strategy for enhancing self-efficacy is applying verbal persuasion. In this study, the researchers encouraged participants who achieved any level of success, however minor, and offered verbal feedback when opportunities for such achievements arose.

#### Physiological states

Information related to physiological states, formed from an individual’s self-evaluation of a specific behavior’s physical and psychological effects, can influence their subsequent judgment of their capabilities and abilities to perform that behavior.

The researcher followed up with the intervention group by phone regarding homework completion. No intervention was administered to the control group. Immediately and 4 weeks after the intervention, the IPV Questionnaire and the Summary of Diabetes Self-Care Activities Measure were completed again by the intervention and control groups. Upon completion of the study, following the ethical standards that govern research, the content of the life skills training was disseminated to the control group in the form of a pamphlet. Moreover, the pamphlet detailed the content of the life skills training and relevant support centers, thus enabling women to access assistance in the event of violence.

### Instruments

The data were collected using the demographic forms, Domestic Violence Questionnaire and Treatment Adherence Questionnaire.

Demographic information form that included three sections was administered: (1) demographic characteristics of the patient (age, duration of marriage, number of marriages, number of children, level of education, etc.), (2) characteristics of the disease (type of disease, history of hospitalization due to the disease, smoking, family history of the disease, etc.), (3) demographic information of the spouse (occupation, age, level of education, etc.) (Table 2).

**Table 2:** Comparison of the demographic variables between the intervention group and the control group

Variable	Variable levels	Intervention group (*n* = 50)	Control group (*n* = 50)	Statistics and significance
		Mean ± SD or *N* (%)	Mean ± SD or *N* (%)	Statistic (***P***-value)
Age		53.12 ± 9.71 ^a^	53.12 ± 9.17 ^a^	-0.069^*^(0.945)
Duration of marriage		33.20 ± 10.84 ^a^	33.34 ± 10.11 ^a^	-0.067^**^(0.947)
Spouse’s age		57.52 ± 9.53 ^a^	56.68 ± 9.81 ^a^	0.434^**^(0.665)
Duration of diabetes		11.60 ± 5.89 ^a^	11.02 ± 6.79 ^a^	-0.863^*^(0.388)
Level of education	Elementary	24(48.0) ^b^	30 (60.0) ^b^	5.074 ^***^(0.083)
	Middle school and high school	19)38.0(^b^	19 (38.0) ^b^	
	University	7 (14.0) ^b^	1 (2.0) ^b^	
Job	Employed	4 (8.0) ^b^	1 (2.0) ^b^	1.895^***^(0.161)
	Unemployed	46 (92.0) ^b^	49 (98.0) ^b^	
Average monthly income	Within the cost	29(58.0) ^b^	33 (66.0) ^b^	1.442^****^(0.537)
	More than the cost	1 (2.0) ^b^	0 (0) ^b^	
	Less than cost	20 (40.0) ^b^	17 (34.0) ^b^	
Spouse’s level of education	Illiterate	5 (10.0) ^b^	1 (2.0) ^b^	5.385^****^(0.250)
	Elementary	14 (28.0) ^b^	23 (46.0) ^b^	
	Middle school	9 (18.0) ^b^	8 (16.0) ^b^	
	High school	15(30.0) ^b^	11 (22.0) ^b^	
	University	7 (14.0) ^b^	7 (14.0) ^b^	
Spouse’s job	Unemployed	1 (2.0) ^b^	1 (2.0) ^b^	1.147^****^(0.947)
	Worker	1(2.0) ^b^	2 (4.0) ^b^	
	Businessman	20 (40.0) ^b^	18 (36.0) ^b^	
	Self-employment	5 (10.0) ^b^	7 (14.0) ^b^	
	Retired	23 (46.0) ^b^	22 (44.0) ^b^	

a: Numbers are Mean ±SD; b: Numbers are N (%); SD= Standard Deviation; N= Number

*Independent t test was performed and values are t

** The conducted Mann−Whitney test and the reported statistic is Z

*** The test performed is chi−squared and values are X2

****The test performed is Fisher’s exact test

#### IPV questionnaire

The domestic violence questionnaire by Tabrizi et al. (2013) was used to measure IPV. This questionnaire has 3 domains: (1) IPV, (2) patriarchal beliefs, (3) traditions and family upbringing, and learning violence. In this study, the IPV domain of this questionnaire was used. This questionnaire contains 61 items, but the section used in this research, which measured the types of IPV, contains 26 items in four domains: *psychological spousal abuse* is measured with 11 items (items 1 to 11), *economic* with 5 items (items 12 to 16), *physical* with 6 items (items 17 to 22), and *sexual* with 4 items (items 23 to 26). The items are scored on a five-point Likert scale, ranging from “always” to “never”. The highest incidence of violence is assigned a score of 5, while a score of 1 is given to “never”. The IPV score ranges from 26 to 130, with a higher score indicating a greater prevalence of IPV. The psychometric evaluation of this questionnaire was performed by Tabrizi et al. in 2013. Face and content validity in this study was examined qualitatively according to the opinion of professors with expertise in the measured concept. Reliability was confirmed by examining internal consistency with Cronbach’s alpha of 0.83 [26].

#### Treatment adherence questionnaire

To measure self-care behaviors in patients, Tobert et al.‘s Summary of Diabetes Self-Care Activities Measure (2000) was used, which includes the status of performing self-care behaviors in five domains of diet, physical activity, self-monitoring of blood sugar, foot care, and medication use in patients with diabetes, using 12 items, in the last seven days. Each item’s response ranges from 0 to 7. The minimum score obtained by the patients is 0, and the maximum score is 84. Higher scores indicate better self-care. This questionnaire has been used in many studies in the world, and its validity and reliability have been confirmed. In Raisi et al.‘s study, the content validity index (CVI) for all items was reported to be 0.8 to 1. Reliability was checked by examining internal consistency, and Cronbach’s alpha was 0.82 [27].

### Statistical analysis

**Fig. 1:**
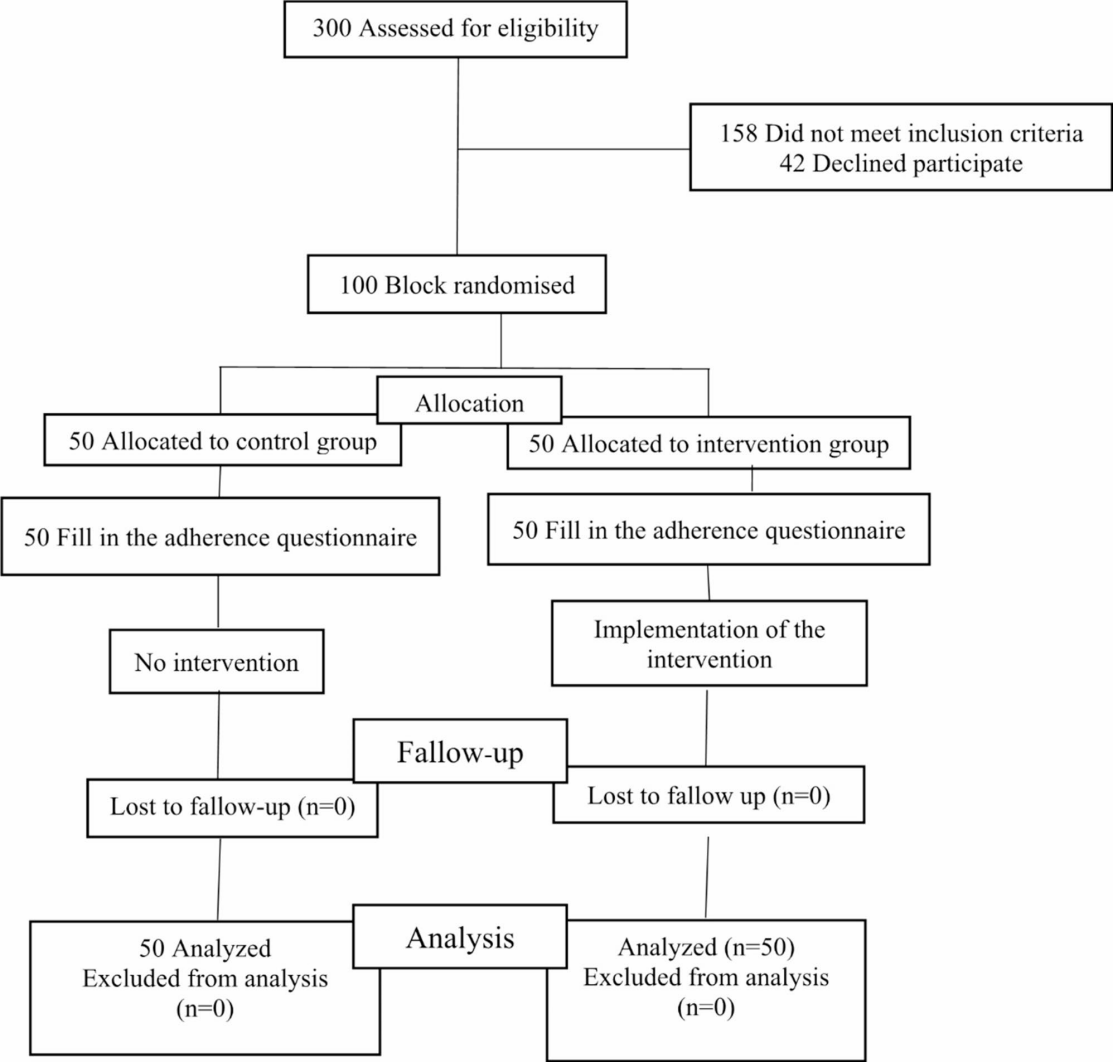
Consort diagram

Intention-to-treat analysis was considered at the start of the trial. In the end, all the participants in each group remained in their respective group. Thus, the intent-to-treat analyses were identical to the per-protocol analyses (Fig. 1). The data were analyzed in SPSS v. 20 software with independent t-tests, Mann-Whitney U, chi-square, Fisher’s exact test, repeated measure analysis of variance, and LSD post-hoc test, and the significance level in all cases was < 0.05. The data were analysed from 18 February 2023 to 10 March 2023.

## Results

The mean (± SD) of age in the intervention and control groups were 53.12 (± 9.71) and 53.12 (± 9.17), respectively. The mean (± SD) of the duration of marriage in the intervention and control groups were 33.20 (± 10.84) and 33.34 (± 10.11), respectively. The two groups did not have statistically significant differences in terms of quantitative and qualitative variables (*P* > 0.05) (Table 2).

The within-group comparison in the intervention group revealed a statistically significant difference in the mean of treatment adherence, total IPV, and psychological, economic, and sexual domains (*P* < 0.001). However, there was no statistically significant difference in the control group (*P* > 0.082). In terms of the physical domain, over time, no statistically significant difference was observed in either group (*P* > 0.05; Table 3).

**Table 3:** Intragroup comparison of mean scores for domestic violence, its domains and treatment adherence

Variable		Time	Group	
			Intervention	Control
			M ± SD	M ± SD
Psychologic violence		Pre-test	24.56 ± 6.29	23.94 ± 6.49
		Post-test	18.48 ± 4.67	23.76 ± 7.24
		Follow-up	17.24 ± 4.71	24.82 ± 6.84
Statistics F (*P*-value)			160.958 (< 0.001)	2.685 (0.082)
Economic violence		Pre-test	6.30 ± 2.67	6.24 ± 2.68
		Post-test	5.60 ± 1.34	6.30 ± 2.79
		Follow-up	5.46 ± 1.07	6.34 ± 2.95
Statistics F (*P*-value)			11.574 (0.001)	0.589 (0.545)
Physical violence		Pre-test	6.46 ± 1.73	6.40 ± 1.65
		Post-test	6.20 ± 0.76	6.48 ± 1.85
		Follow-up	6.16 ± 0.65	6.54 ± 1.89
Statistics F (*P*-value)			3.610 (0.062)	2.958 (0.079)
Sexual violence		Pre-test	6.34 ± 2.09	6.10 ± 1.94
		Post-test	5.00 ± 1.28	6.08 ± 1.72
		Follow-up	5.06 ± 1.33	6.42 ± 1.82
Statistics F (*P*-value)			62.795 (< 0.001)	3.847 (0.042)
Total violence		Pre-test	43.66 ± 9.45	42.68 ± 9.93
		Post-test	35.28 ± 6.03	42.62 ± 10.42
		Follow-up	33.92 ± 5.85	44.12 ± 10.32
Statistics F (*P*-value)			130.718 (< 0.001)	4.868 (0.013)
Treatment adherence	Pre-test		23.94 ± 6.49	24.56 ± 6.29
	Post-test		23.76 ± 7.24	18.48 ± 4.67
	Follow-up		24.82 ± 6.84	17.24 ± 4.71
Statistics F (*P*-value)			2.243 (0.120)	195.897 (< 0.001)

M: Mean, SD: Standard deviation

The pairwise comparison of time in terms of psychological and economic violence showed that in the intervention group, the average score from pre-test to follow-up decreased, and the difference between post-test and pre-test, follow-up and pre-test, and follow-up and post-test was statistically significant. Nevertheless, the control group showed no significant difference from the three groups. Physical violence in the intervention and control groups had no significant difference between the three groups (*P* > 0.05). The pairwise-time comparison in terms of treatment adherence indicated that in the intervention group, the average score from pre-test to follow-up decreased; besides, the difference between post-test and pre-test, follow-up and pre-test, and follow-up and post-test was statistically significant. Meanwhile, the control group showed no significant difference from the three groups (Table 4).

**Table 4:** Comparison between the two intervention and control groups of mean scores for change in IPV, its domains, and treatment adherence from pre-intervention to one-month post-intervention

Variable	Time difference	Group		t (***P***-value)	95% Confidence interval
		Intervention	Control		
		Mean ± SD	Mean ± SD		
Psychologic violence	T2-T1	-6.08 ± 2.21	-0.18 ± 2.69	-11.972 (< 0.001)	-6.88; -4.92
	T3-T1	-7.32 ± 3.89	0.88 ± 3.63	-10.902 (< 0.001)	-9.69; -6.71
	T3-T2	-1.24 ± 2.93	1.06 ± 3.94	-3.311 (< 0.001)	-3.68; -0.92
Economic violence	T2-T1	-0.70 ± 1.39	0.06 ± 0.59	-3.565 (0.001)	-1.18; -0.34
	T3-T1	-0.84 ± 1.75	0.10 ± 0.74	-3.495 (0.001)	-1.4; -0.40
	T3-T2	-0.14 ± 0.50	0.04 ± 0.64	-1.576 (0.118)	-0.41; 0.05
Physical violence	T2-T1	-0.26 ± 0.99	0.08 ± 0.34	-2.305 (0.025)	-0.63; − 0.05
	T3-T1	-0.30 ± 1.09	0.14 ± 0.53	-2.557 (0.012)	-0.78; -0.10
	T3-T2	-0.04 ± 0.20	0.06 ± 0.31	-1.907 (0.060)	-0.20; 0.001
Sexual violence	T2-T1	-1.34 ± 1.06	-0.02 ± 0.62	-7.585 (< 0.001)	-1.67; -0.97
	T3-T1	-1.28 ± 1.18	0.32 ± 1.24	-6.624 (< 0.001)	-2.08; -1.12
	T3-T2	0.06 ± 0.47	0.34 ± 0.96	-1.851 (0.068)	-0.58; 0.02
Total violence	T2-T1	-8.38 ± 4.06	-0.06 ± 3.09	-11.544 (< 0.001)	-9.75; -6.89
	T3-T1	-9.74 ± 5.95	1.44 ± 4.21	-10.844 (< 0.001)	-13.23; -9.13
	T3-T2	-1.36 ± 3.47	1.50 ± 4.14	-3.744 (< 0.001)	-4.38; -1.34
Treatment adherence	T2-T1	11.40 ± 4.23	0.68 ± 3.49	13.817 (< 0.001)	9.18; 12.26
	T3-T1	14.08 ± 6.48	-0.16 ± 2.97	14.006 (< 0.001)	12.23; 16.26
	T3-T2	2.68 ± 5.06	-0.86 ± 2.43	4.443 (< 0.001)	1.95; 5.12

SD: Standard deviation / Test performed: independent t test/ T1: Pre−test Time/ T2: Posttest Time / T3: Fallow−up Time

Comparing the average score changes between the groups showed that the mean score of changes in psychological violence in all three times had a statistically significant difference between the two groups. Nevertheless, the average score of changes in economic, physical, and sexual violence, except in the follow-up and post-test stages, showed a statistically significant difference between the two groups on two other occasions. The comparison of the average scores between the groups revealed that the average scores of treatment adherence change between pre-test and post-test in the intervention and control groups were increasing and significantly higher in the intervention group than the control group. Besides, the difference between pre-test and follow-up, and between post-test and follow-up, was positive in the intervention group (increase in adherence) and negative in the control group (decrease compared to the previous time). Moreover, the two groups showed statistically significant differences (Table 5).

**Table 5:** Post hoc comparison of domestic violence, its domains and treatment adherence (two times compared)

Variable	Group	Time a	Time b	Mean difference (a - b)	Standard error (***P***-value)	95% confidence level for the mean difference
Psychologic violence	Intervention	T2	T1	-6.08	0.313 (< 0.001)	-6.71; -5.45
		T3	T1	-7.32	0.550 (< 0.001)	-8.43; -6.21
			T2	-1.24	0.415 (0.004)	-2.07; − 0.41
	Control	Lack of significance in the comparison of three groups				
Economic violence	Intervention	T2	T1	-0.70	0.20 (< 0.001)	-1.09; -0.31
		T3	T1	-0.84	0.25 (< 0.001)	-1.34; − 0.34
			T2	-0.14	0.07 (0.051)	-0.28; 0.00
	Control	Lack of significance in the comparison of three groups				
Physical violence	Intervention	Lack of significance in the comparison of three groups				
	control	Lack of significance in the comparison of three groups				
Sexual violence	Intervention	T2	T1	-1.34	0.15 (< 0.001)	-1.64; -1.04
		T3	T1	-1.28	0.17 (< 0.001)	-1.61; -0.95
			T2	0.06	0.07 (0.371)	-0.07; 0.19
	Control	T2	T1	-0.02	0.09 (0.821)	-0.20; 0.16
		T3	T1	0.32	0.17 (0.073)	-0.03; 0.67
			T2	0.34	0.14 (0.016)	0.07; 0.61
Total violence	Intervention	T2	T1	-8.38	0.57 (< 0.001)	-9.53; -7.23
		T3	T1	-9.74	0.84 (< 0.001)	-11.43; -8.05
			T2	-1.36	0.49 (0.008)	-2.35; -0.37
	Control	T2	T1	-0.06	0.44 (0.891)	-0.94; 0.82
		T3	T1	1.44	0.60 (0.019)	0.24; 2.64
			T2	1.50	0.59 (0.014)	0.32; 2.68
Adherence to treatment	Intervention	T2	T1	11.40	0.599 (< 0.001)	10.20; 12.60
		T3	T1	14.08	0.917 (< 0.001)	12.24; 15.92
			T2	2.68	0.716 (< 0.001)	1.24; 4.12
	Control	Lack of significance in the comparison of three groups				

Test performed: LSD follow−up test/ T1: Pre−test Time/ T2: Posttest Time / T3: Fallow−up Time

## Discussion

This study aimed to determine the effect of life skills training based on self-efficacy theory on IPV and adherence to treatment in women with diabetes experiencing IPV. The findings indicated that life skills training based on self-efficacy theory was associated with a reduction in IPV and an improvement in treatment adherence. However, in the control group, no change was observed regarding IPV and adherence to treatment. The researcher could not find any study on the available websites where the research population was women experiencing violence with T2DM; consequently, the results were compared with studies that taught some aspects of life skills or were based on self-efficacy theory.

​ The results of the study by Babaheidrian et al. (2021) [28] were in line with the results of the present study. However, the findings were different from the results of Mirahmadian et al. (2001) [29] and Mohammadbeigi et al. These studies revealed that teaching problem-solving skills [29], anger control skills [30], and life skills [24] was not effective for women under IPV. The reason for the difference in the findings from the present study can be due to the research population and the method of presenting the educational content. These studies were conducted on women under violence without chronic diseases. Mirahmadian et al. and Mahmoudian et al. taught only two (i.e., problem-solving skills and anger control skills) of the 10 parts of the life skills training content presented in the present study. People face different challenges in life [31]; By learning and practicing life skills, people can accept the responsibilities and duties related to their social role without harming themselves or others and face the challenges and problems of daily life effectively. These skills help to deal effectively with conflicts and situations in life, so that one can act positively and adaptively in relation to other people, society, culture, and environment [20].

The mentioned results demonstrate that using self-efficacy theory strategies in women subjected to IPV has led to a notable reduction in IPV. Therefore, it is inferred that self-efficacy strategies (including mastery experiences, vicarious experiences, verbal persuasion, and emotional and physiological states) are effective in educational interventions. The use of behavior change models and theories increases the probability of intervention effectiveness and helps to change behavior by identifying individual and environmental characteristics that somehow affect behaviors [32]. Taqdisi et al. (2014) reported a significant reduction in violence after an educational intervention based on self-efficacy theory [33]. The training program, based on the self-efficacy theory, prompted participants to engage in introspective reflection on their personal characteristics. This encouragement enabled them to identify their strengths and weaknesses, enhance their acceptance and compromise, increase their belief in their capabilities, and improve the performance of adaptive positive behaviors. Furthermore, implementing face-to-face and group training yielded a notable positive impact on the outcomes of this research. These training modalities assisted participants in reducing mental pressure and enhancing their capacity to accept reality and cope with it effectively [34]. Furthermore, the participants were able to identify with the characteristics of the group and gain a deeper understanding of the issues they were facing. Group participation provides a unique opportunity to identify novel solutions through exchanging ideas with other group members [31, 34], which is not feasible in an online training environment and not in an online setting [29].

The results showed the lack of effect of the educational intervention on physical violence in the intervention group. The average score of physical violence in the pre-test stage was low; the intervention reduced physical violence but could not create a significant difference. Thus, it is suggested that studies test the effectiveness of this educational intervention in physical violence in other populations with chronic diseases.

The results indicated that in the control group, changes in sexual violence increased between pre-test and follow-up, and the difference was statistically significant. The reason for the significant difference in sexual violence could be the lack of awareness and familiarity of the participants in the control group with the concepts and examples of sexual violence; after completing the questionnaire in the pre-test stage, they were introduced to examples of sexual violence and reported more exposure at later stages when completing the questionnaire and, therefore, a higher exposure rate was reported [35].

The mean changes in the treatment adherence score demonstrated a statistically significant difference between the two groups at three-time points. The results of the studies conducted by Oraki and Isazadeh (2021) [34], Eshghi Mutlaq et al. (2018) [35], and Zamani Alawijeh et al. (2018) [36] were found to be consistent with those of the present study.

The study of Torabzadeh et al. (2016) on the effect of problem-solving technique on T2DM patients with cognitive impairment showed that problem-solving skill training only caused a significant difference in three-month blood sugar and HDL in the within-group comparison, but fasting blood sugar, triglyceride, total cholesterol, LDL, physical activity, and drug intake were not significantly different between the intervention and control groups. The reason for the difference in the findings could lie in the research population and the type of intervention provided. The study population of Torabizadeh had mild to moderate cognitive impairment, while in the present study, diabetic women with experience of IPV who did not suffer from cognitive impairment participated. Hill-Briggs and Gamel (2007) found in their review study that problem-solving skills only improved three-month blood sugar in 50% of adults and 25% of children. Other skills also play a significant role in treatment adherence behaviors. For example, some studies found that lack of time is an obstacle to the patient’s physical activity [37, 38], and time management is very important, especially for women who are faced with multiple responsibilities and roles and have little time to follow therapeutic care regimens [37]. Because the adherence process in diabetes is time-consuming, diabetes education and care professionals estimate that routine adherence to recommended care regimens takes approximately two hours per day, and more time is needed for people who are newly diagnosed or have more care needs [39]. Therefore, the ability to manage time is a skill that can affect the successful implementation of treatment adherence behaviors [37]. According to the results of the present study, people suffer from stress due to chronic disease and its unpredictable course. Anger, anxiety, and fear caused by chronic diseases such as diabetes and its complications cause physical and mental exhaustion, and this plays an effective role in disappointment with the treatment and non-adherence to the recommended diets [34, 40–42]. Thus, if the person is also subject to violence, this problem will become an additional cause. Teaching life skills based on the theory of self-efficacy causes self-efficacy. by providing a model, creating motivation, and following up training in following the treatment of patients [43]. Since self-efficacy is an important prerequisite for behavior change, people who feel more self-efficacy perceive their health status better and more positively and pay attention to self-care behaviors in diabetes [44]. To more precisely prove the effectiveness of the current intervention, it is suggested that future studies objectively measure the adherence of women with diabetes under IPV by examining fasting blood sugar and three-month blood sugar so that a more accurate judgment can be made.

This research has some limitations. First, the presence of anxiety among the female participants in the study when completing the IPV questionnaire may have resulted in an underestimation of negative constructs and an overestimation of positive constructs. Therefore, it would be prudent to exercise caution when generalizing the findings of this research. A second limitation is the brief follow-up period may have contributed to the absence of a discernible impact of the intervention on the physical dimension. In the present study, adherence to the treatment was determined through self-report, which may have led to an overestimation of adherence. It is recommended that future studies utilize objective measurements, such as FBS or Hb A1C, to assess treatment adherence. The present study was conducted in a single city in southern Iran. It is important to note that a number of factors, including cultural, economic, and social conditions, can influence the occurrence of IPV. It is thus imperative that the results of this study be generalized with due caution.

In order to ensure that the results are more effective over a longer period of time, it would be beneficial if some studies collected data on violent spouses. In this respect, the ethical and safety recommendations of the WHO for interventional research on violence against women stipulate that women in the control group should also receive some form of support. However, the study merely presented the content of life skills training and relevant support centers in a pamphlet.

## Implications for research and practice

The present study offers empirical evidence to support the broader implementation of life skills training to enhance diabetes management adherence among women experiencing IPV. In treating chronic patients, healthcare providers must consider the issue of IPV and the medical issues related to the disease and its treatment and care. This consideration could impact the process of disease management and patient adherence. It is recommended that managers and planners of the healthcare system adopt an interprofessional approach to treating and managing chronic diseases such as diabetes in women. In addition to diagnosis, they provide appropriate solutions such as teaching patients life skills based on self-efficacy theory and improving patient adherence. It is suggested that a similar study be conducted on women under violence with other chronic diseases in different communities with longer follow-up periods.

## Conclusion

The findings of the present study demonstrate that a life skills training program based on self-efficacy theory is an effective approach for reducing IPV and enhancing treatment adherence among women with diabetes who have experienced IPV. The implementation of a life skills training program based on this theory can facilitate the resolution of interpersonal conflicts and reduce the occurrence of negative behaviors. It is therefore recommended that healthcare providers employ life skills training in conjunction with other interventions to reduce intimate partner violence and subsequently enhance treatment adherence in other patients with chronic diseases. Given the reciprocal nature of violence, it would be beneficial for future studies to administer a similar intervention to the male partners of the participants, in addition to the female partners.

## Data Availability

All results are available in the text of the manuscript and Tables. The datasets used during the current study are available from the corresponding author on reasonable request.
